# Drone Model Classification Using Convolutional Neural Network Trained on Synthetic Data

**DOI:** 10.3390/jimaging8080218

**Published:** 2022-08-12

**Authors:** Mariusz Wisniewski, Zeeshan A. Rana, Ivan Petrunin

**Affiliations:** Digital Aviation Research and Technology Centre (DARTeC), Cranfield University, Cranfield MK43 0FQ, UK

**Keywords:** unmanned aerial vehicles, drones, airport security, convolutional neural network, synthetic images, synthetic data, domain randomization, drone detection, drone classification, drone identification, artificial intelligence

## Abstract

We present a convolutional neural network (CNN) that identifies drone models in real-life videos. The neural network is trained on synthetic images and tested on a real-life dataset of drone videos. To create the training and validation datasets, we show a method of generating synthetic drone images. Domain randomization is used to vary the simulation parameters such as model textures, background images, and orientation. Three common drone models are classified: DJI Phantom, DJI Mavic, and DJI Inspire. To test the performance of the neural network model, Anti-UAV, a real-life dataset of flying drones is used. The proposed method reduces the time-cost associated with manually labelling drones, and we prove that it is transferable to real-life videos. The CNN achieves an overall accuracy of 92.4%, a precision of 88.8%, a recall of 88.6%, and an f1 score of 88.7% when tested on the real-life dataset.

## 1. Introduction

Drones pose a risk to the security of infrastructures such as airports, prisons, and crowded areas such as stadiums. A drone intrusion affected approximately 1000 flights at Gatwick airport in 2018. In Britain, drones are reportedly used to deliver drugs and mobiles to prisoners. There are also numerous reports of drones flying over crowded stadiums at events. To prevent such unwanted intrusions, detection systems are required to monitor the airspace around objects of interest.

Counter drone technologies consist of prevention, detection, and mitigation systems. Prevention aims to stop drones from flying within a certain area, detection aims to find if any drones exist in a given area, and mitigation aims to incapacitate a drone once its position is known. The detection aspect is particularly challenging because drones are small, the area where they can fly is large, and they share their airspace with other objects (such as birds). Furthermore, it is important to classify the drone to understand the threat level that it poses. The size, model type, and payload (if any) are all of interest to operators of security systems, and access to this information would enable them to provide a more appropriate response. For example, a large drone carrying a suspicious payload may require a more urgent response than a small drone with only a camera attached.

This paper investigates drone model classification; a niche, mostly unexplored, but important part of visual drone classification. In particular, three popular drone models are classified using a convolutional neural network (CNN). This is achieved by creating a synthetic dataset to train the neural network and testing its performance on a real-life dataset of flying drones. It is an extension study of [1] that examines more closely the effects of synthetic noise, dataset size, and simulation parameters (ablation study). It also updates on the latest literature in the related field of drone detection.

The novel contribution presented in this paper is the CNN that is trained on a purely synthetic dataset and can accurately (90%+) predict the model of the drone in a real-life video feed. From the literature, it is understood that previous CNN models used to identify drones have mostly been trained on real-life images. The principal novelty of the work presented here is that our CNN is solely trained and verified using a 3D model of the drone reducing the time cost associated with classifying other drone models in the future. A big focus of this research is bridging the synthetic to real-world gap. Some publications also make use of synthetic images; however, in contrast to those, we test our results on an open-source real-world dataset. To the best of the author’s knowledge, such a CNN, trained on a purely synthetic dataset and verified on an open-source dataset of real drones, has not been presented in the literature.

Commercial detection systems most commonly consist of the following sensors [2]:

- Acoustic (6%);
- Radio Frequency (RF) (26%);
- Radar (28%);
- Visual (40%).

The viability of different sensors and their operating range is evaluated [3]. It is found that, for a drone with a radar cross-section between 0.05 to 0.2 m^2^, humans can see the drone up to 200 m, hear it up to 300 m, infrared sensors are viable up to about 350 m, optical cameras up to about 2500 m and standard X-band radar up to 3000 m.

Acoustic sensors are the least used type of sensor for drone detection. They have a relatively short range compared with other sensors but have certain advantages such as being able to operate in the dark. An acoustic system presented by Kolamunna et al. [4] correctly detects and identifies drone models using a neural network trained on the sound of each drone. Another system presented by Liu et al. [5] uses a hybrid system of visual cameras and microphones to detect drones. They find that using the audio improves detection accuracy. Ciaburro et al. [6] performs a study to detect a flying drone indoors. Iannace et al. [7] expands on it by using logistic regression, and reports a drone detection accuracy of 99.4% at distances of up to 9 m in a noisy environment. Jamil et al. [8] use a combination of audio and visual features to detect drones, thunderstorms, birds and planes. They report an accuracy of 98.5% overall.

RF methods look at the RF signature of the drone. They can be cheaper than other sensors, such as the optical cameras, and can perform at long range. However, they cannot detect autonomous drones that have no RF transmissions. A hierarchical classifier that detects the presence of a drone is presented by Nemer et al. [9]. It can identify three models (Parrot Bebop, Parrot AR, DJI Phantom), and for the Parrot model, it is able to identify the flight mode of the drone. The DronEnd system [10] is used to jam the RF of the drones. Yang et al. [11] are able to detect drones with close to 100% accuracy at distances up to 2.4 km. Al-Sa’d et al. detect different types of drones at different flight modes such as on, off, connected, hovering, flying, and video recording. By using a deep neural network, they record a 48.6% accuracy for detecting the presence, drone type, and the flight mode. They report a 99.7% accuracy for detecting the presence of the drone only.

Holographic radars [12] can detect and classify 1 m${}^{2}$ drones at a range of 20 nmi. A CNN [13] can correctly classify drones and non-drones at an accuracy of 98.9%. Multistatic radars can be used to classify loaded and unloaded drones [14] by use of micro-Doppler analysis. Similarly, micro-Doppler signatures can be used to predict birds from drones [15]. A CNN trained to classify the micro-Doppler images can identify the drone models [16].

There exist numerous attempts at detecting drones using visual methods. Demir et al. [17] show a system that can detect drones as far as 700 m away in real-time. To achieve this, they use a matrix of static cameras and the background subtraction method. Another system presented by Seidaliyeva et al. [18] also uses background subtraction to detect drones. They then use a CNN to classify the detected object into drone, bird, and background classes. Zhang et al. [19] compare detection neural network models (Faster R_CNN, Retina net, SSD, and their own), to detect drones in infrared videos.

Datasets containing videos and images of drones, labelled with the ground truth position of the drone in the image, are crucial to the development of drone detection methods. The Anti-UAV challenge [20] provides datasets of visual and thermal video feeds and challenges researchers to detect the drones in the videos. In the test dataset, the position and the size of the drone in each frame are provided. Bird vs. Drone Challenge [21] provides another dataset of videos containing drones and birds, with the position and the size of the drone provided in each frame. To try and unify the datasets of flying drones, a benchmark aims to directly compare drone detection methods [22]. Furthermore, to test the re-identification of drones in multiple-camera environments, another benchmark is proposed [23].

Detection of drones in videos has been tried in literature. Siam R-CNN [24] reports the best precision score of 95.70% on the infrared Anti-UAV dataset. The best submission to the drone vs. bird challenge [25] uses a trained ResNet110v2 CNN to classify the flying objects into drone, bird, and none categories. Fan et al. [26] use Faster-RCNN to detect drones in images. CNN and Generic Fourier Descriptor (GFD) classifiers [27] are used to identify birds and drones in images. YOLOv4 is used to detect and classify flying objects into bird, helicopter, and multirotor classes [28] with a reported accuracy of 83% and mean average precision of 84%. Furthermore, some publications attempt to identify drone models. A drone identification system presented by Lee et al. [29] uses 2000 images gathered from the internet to train a CNN to identify drone models. YOLOv3 is used to detect and classify whether the drone is a tricopter, a quadcopter or a hexacopter [30]. Lastly, YOLOv2 is used to detect and classify whether a drone is carrying a payload [31].

Using synthetic datasets for drone classification has been attempted in the literature. The advantage of using a synthetic dataset is that the ground truth is known, and it is possible to generate a larger and more varied dataset, by the process of domain randomization. This method is also quicker compared to producing real-life datasets, where the ground truth must be manually labelled. A method of generating synthetic drones is shown in [32]. It is done by overlaying 3D drone models on top of random backgrounds. Another method shown by Peng et al. [33] improves this by using a Physically Based Rendering Toolkit to generate photorealistic images of drones. Ref. [34] uses synthetic images of DJI Mavic and DJI Inspire generated using Unreal Engine to train a neural network that segments the parts of the drone: the body, motors and the camera. It then predicts the orientation—pitch, roll and yaw—of the drone. Lastly, it identifies the model of the drone. To get around the problem of scarce drone image data, Ref. [35] uses generative adversarial networks and topological data analysis to improve the accuracy of a drone detection neural network.

Synthetic images are commonly used for the detection of objects other than drones. Tremblay et al. [36] detect cars on the real-life KITTY dataset by training a neural network on a synthetic dataset. They also apply domain randomization, which is the process of randomizing parameters of the simulation. The aim of this practice is to improve the ability of the neural network to correctly learn the features of the object. A deep neural network trained used to control a robot arm based on visual input [37] is a successful application of transferring a model trained on purely synthetic data to a real-world application. Ward et al. [38] use synthetic images to classify different types of ships from overhead satellite imagery. To achieve the best results, they mix real-world and synthetic data in their training dataset.

From the literature, we identified the gap of identifying drone models in images, with very few publications attempting it. Most publications in this space focus on the drone vs. bird problem, which is very important. However, the identification of the drone model is also important as it can provide a threat level estimate to security operators. For example, a larger drone poses a bigger threat than a smaller drone because it can carry a larger payload. We believe that correctly identifying the drone model is an initial step in the much larger picture of identifying the potential threat level of the drone. The method presented in this paper uses synthetically generated drones to train a CNN to detect drones in a real-life dataset. By showing this method is viable, it opens the possibility to further develop it to detect other types of drones or flying objects in the future. Most importantly, this method of synthetically generating drone images has advantages over creating a real-life dataset, as it reduces the costs associated with the dataset creation—for example, the labelling of the bounding boxes on the images is fully automated.

This paper is divided into five sections: In Section 2, the methodology of generating synthetic images is described. This includes the explanation of a rendering script used to generate synthetic images in Blender, and an explanation of domain randomization, the process of varying simulation parameters. In Section 3, the training and tuning of the CNN using synthetic images are discussed, and the results are presented. In Section 4, the results of the performance of the CNN when tested on a real-life dataset are discussed. The results are compared to other results reported in literature. In Section 5, the findings are concluded, the shortcomings of the study are discussed, and further work is suggested.

## 2. Methodology for Dataset Generation

In this section, we explain the process of creating the synthetic dataset using Blender https://www.blender.org/ (accessed on 27 March 2022) an open-source 3D modelling program. We use it to create a simulation where the camera takes images of the drone with randomized image parameters. For example, the position, the orientation of the drone, and the background are randomized in order to create photorealistic images of the drone. After each step, an image of the drone is generated. After this, the parameters—the position, orientation, and background—are randomized to create another image. In theory, a practically infinite dataset size could be generated this way.

The development of the dataset is a challenge with training neural networks. Drawing bounding boxes around the target is a time-intensive task when creating a real-life dataset. Advancements in synthetic image generation by using domain randomization such as [36,37] show that it is possible to train a neural network model on synthetic data and use it on real-life problems. Hence, we decided to train a model on a purely synthetic dataset, and test it on a real-life video feed of the Anti-UAV dataset [20].

The advantage of this approach is:

- Time and monetary cost savings on performing a real-life experiment involving the drones. The drones themselves are expensive and require trained operators and a camera operator to follow the drones;
- Time saving because of automated labelling. In real-life videos, each frame has to be manually labelled;
- Larger variability in the dataset. It is possible to create niche scenarios that are hard to create in real life. For example, conditions such as rain or snow can be created in the simulation. These conditions pose challenges when creating real-life experiments involving drones. Although we do not create such scenarios here, it is an interesting idea for future study.

**Figure 1:**
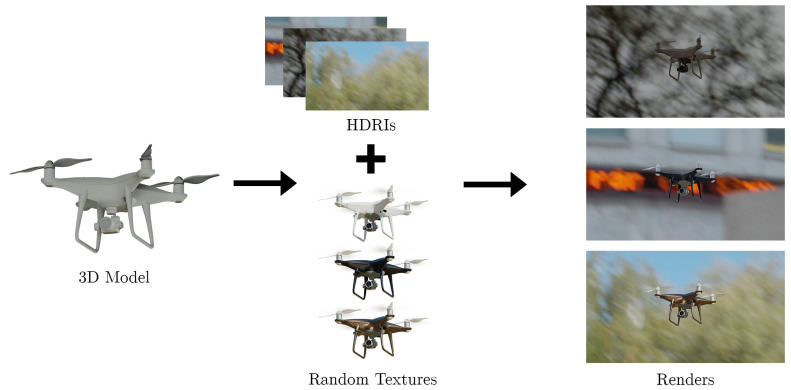
Process of rendering a synthetic drone. A 3D model of the drone is required and in this example we show a DJI Phantom. Random textures are applied to the 3D model. A high dynamic range image (HDRI) containing a realistic lighting setup is set as the background scene. The drone is rendered using the Cycles engine in Blender.

**Figure 2:**
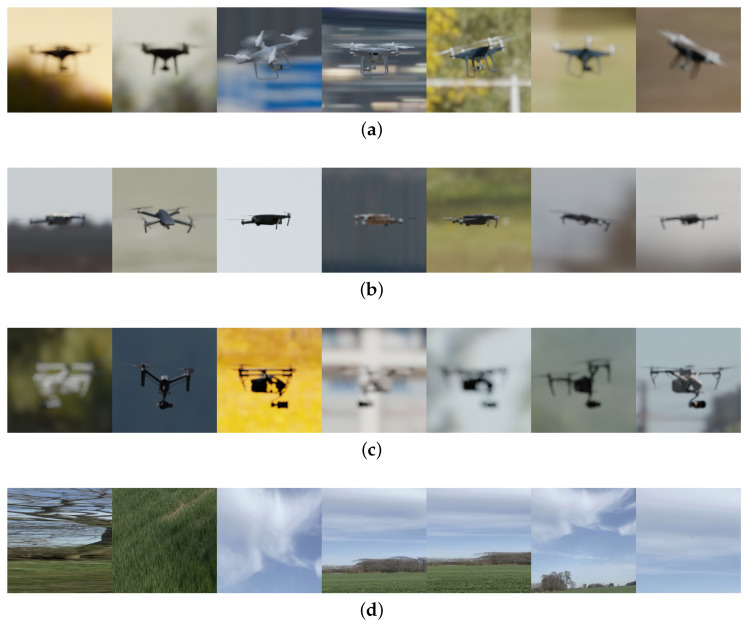
The synthetic drone images with all of the randomizations enabled, generated by the simulation in Blender, used for training and validation of the convolutional neural network (CNN). The images are intended to cover a large number of potential scenarios, with different background and lighting conditions. The camera parameters such as the focal length and focus point are also randomized to make the dataset as photorealistic as possible. The textures of the drones are also randomized with the aim of training the CNN to detect the shape, instead of the colour of the drone. Thus, the colour of the drones appears different in some of the images even for the same drone models. (**a**) DJI Phantom, (**b**) DJI Mavic, (**c**) DJI Inspire and (**d**) No Drone.

To create the dataset, we modelled DJI-Phantom and DJI-Mavic, and we found a free 3D model of DJI-Inspire https://sketchfab.com/3d-models/dji-inspire-2-with-zenmuse-x5s-3979efe28b3a4221bdd462638582d0a6/ (accessed on 27 March 2022) online. We then created a script to generate random images of the drone. The process of image generation is shown in Figure 1. It shows how the images are rendered by taking the 3D model of a drone and adding textures and high dynamic range images (HDRI). The synthetic images with all of the randomizations enabled are shown in Figure 2. The figure shows renders generated by the simulation created in Blender. Different drone types: DJI Phantom, DJI Mavic, and DJI Inspire are shown. Lastly, the no drone class is shown, which does not contain any drone images, and only contains random background images. These images are used to train and validate the neural network in Section 3.

### 2.1. Rendering Script

To generate the synthetic dataset, a Python script is used within Blender to randomize certain parameters of the simulation. The drone is animated to take off from the origin and fly around for 200 frames. During each animation frame, the position of the camera is randomized and within 200 m of the drone. The camera is programmed to always point towards the drone. After 200 frames, the animation is complete. The animation is reset, the HDRI is changed, and the texture of the drone is updated. The HDRI contains a background image for the scene, as well as a realistic lighting setup. The HDRIs were acquired from PolyHaven https://polyhaven.com (accessed on 27 March 2022).

Algorithm 1 shows the pseudocode for the rendering script. At the start, the position, focal length and focal distance limits are defined. The number of animation frames is set—this depends on the length of the animation of the drone. FollowDrone is set to true—in the program, this sets the camera to automatically follow the drone. The program then enters a while loop of 10 iterations. Every iteration, the texture and the HDRI are randomized. Then, the animation is started. Every animation frame, the position of the camera is randomized within the XYZ position limits. The focal length and focal distance are also randomized. The frame is then rendered and cropped to the size of the drone. Lastly, the image is saved. In total, 1000 images are generated, with 10 different backgrounds and textures.
**Algorithm 1** Synthetic drone rendering scriptXYZPositionLimits = [Xmin, Xmax, Ymin, Ymax, Zmin, Zmax]FocalLengthLimits = [FLmin, FLmax]FocusDistanceLimits = [FDmin, FDmax]AnimationFrames = 100FollowDrone = True*i* = 0**while***i* < 10 **do**    randomizeTextures()    randomizeHDRI()    CurrentFrame = 0    **while** CurrentFrame < AnimationFrames **do**        randomizeCameraPosition(XYZPositionLimits)        randomizeFocalLength(FocalLengthLimits)        randomizeFocusDistance(FocusDistanceLimits)        Frame = renderFrame()        CroppedFrame = cropImageToSizeOfDrone(Frame)        saveImage(CroppedFrame)        CurrentFrame += 1    **end while****end while**

### 2.2. Domain Randomization

Preliminary training and testing trials showed that the model translated poorly to the real-world dataset during testing. We hypothesised that this could be because the real-world dataset differs considerably from the synthetic images. In their synthetic-to-real transfer, Tremblay et al. [36] applied unrealistic textures to their 3D cars. The aim of this was to train the neural network about the features of the object, as opposed to the colour or texture. Another thing that we noticed was that the real-life images from the Anti-UAV dataset are not perfectly focused on drones. To counter these effects, we replicated the randomization of textures in our simulation. As shown in Algorithm 1, the texture of the main body of the drone is randomized every 200 frames. The textures were acquired from ambientCG https://ambientcg.com/ (accessed on 27 March 2022). To model the real-world effects of imperfect focus on the drones, the focus point of the camera is randomized every frame. Lastly, a no drone class shown in Figure 2d is added, containing random images of the background. The aim of adding this class is to obtain better differentiation between drone and background images. However, we do not explicitly test the accuracy of this no drone class on the real-life dataset.

To further understand the effects of each of the domain randomization parameters, an ablation study is completed in Section 3.6.

## 3. Convolutional Neural Network Training, Tuning and Results

In this section, we describe the process of training the neural network using the synthetic image dataset generated in Section 2. The aim of this process is to use the CNN to predict the drone models in real life. Thus, we test the performance of the neural network on a real-life dataset of images. We explore the effects of freezing layers and adding synthetic Gaussian noise to the training dataset. We explore the use of different CNN architectures and using different hyperparameters. We perform an ablation study to explore the effect of domain randomization parameters on the performance of the CNN. Finally, we perform a dataset size study. All of the neural networks are coded in Python using the PyTorch library [39]. We train the models on a HPC with a Nvidia A100 graphic card. The CNNs in the following section are trained on a dataset of randomly generated 1000 synthetic images, some of which are shown in Figure 2.

Unless otherwise stated, the CNNs are pre-trained on ImageNet [40], and the hyperparameters shown in Table 1 are used. A learning rate of 0.0001, a momentum of 0.9 [41], and a batch size of 32 is used. The CNN is trained for 100 epochs. A dropout of 0.25 is applied, to prevent from overfitting [42]. The validation is performed on a separate dataset of randomly generated 1000 synthetic images.

To measure the performance of the classification, average accuracy,

(1) $$\frac{\sum_{i=1}^{l}\frac{tp_{i}+tn_{i}}{tp_{i}+fn_{i}+fp_{i}+tn_{i}}}{l}$$

average precision,

(2) $$\frac{\sum_{i=1}^{l}\frac{tp_{i}}{tp_{i}+fp_{i}}}{l}$$

recall,

(3) $$\frac{\sum_{i=1}^{l}\frac{tp_{i}}{tp_{i}+fn_{i}}}{l}$$

and f1 score,

(4) $$2\cdot \frac{\mathrm{precision}\cdot \mathrm{recall}}{\mathrm{precision}+\mathrm{recall}}$$

are calculated [43]. $tp_{i}$ is the true positive value for class *i*, $tn_{i}$ is the true negative value for class *i*, $fp_{i}$ is the false positive value for class *i*, $fn_{i}$ is the false negative value for class *i*, and *l* is the number of classes.

### 3.1. Testing the Neural Network

The CNN is trained and validated on synthetic images of drones. The aim of this work is to use the CNN to correctly predict drone models on real-life images. Hence, a real-life dataset of drones is required to test it. The Anti-UAV dataset [20] contains videos of different drone models flying in real-life. The Anti-UAV dataset contains videos and ground truth labels for each frame containing the drone. The dataset contains different drone models: DJI Inspire, DIJ Mavic-Pro, DJI Phantom, DJI Mavic-Air, DJI Spark, and Parrot drones. Two video feeds are provided, one from a visual camera, and another from a thermal camera. Some of the scenarios provided are filmed at night. To test our CNN, we only use daytime videos from the visual camera. Four daytime videos of each of DJI Phantom, DJI Mavic, and DJI Inspire are selected for the testing of our CNN. We use the provided ground truth label to extract an image of the drone from the video feed. This image of the drone is input into the neural network to predict the drone model. The videos used for testing are:

- 20190926_130341_1_1 (phantom);
- 20190926_130341_1_3 (phantom);
- 20190926_130341_1_4 (phantom);
- 20190926_130341_1_6 (phantom);
- 20190926_142435_1_3 (mavic);
- 20190926_141816_1_5 (mavic);
- 20190926_141816_1_1 (mavic);
- 20190926_144550_1_2 (mavic);
- 20190925_130434_1_4 (inspire);
- 20190925_130434_1_7 (inspire);
- 20190925_131530_1_1 (inspire);
- 20190925_131530_1_4 (inspire).

The images in the dataset vary in quality. For example, in some of the images, the camera is focused on the drone, and it appears sharp. In others, the drone appears blurry, possibly due to the movement of the drone or the camera. Certain artefacts, such as white and black lines, also make the classification task challenging. Extracts from the dataset can be seen in [1,20].

### 3.2. Freezing Layers

For synthetic to real transfer, Ref. [44] found that freezing the layers during the training of the neural network improved the precision. Contrary to this, Ref. [36] found that full learning, without freezing the layers, improved performance. To investigate the effects of freezing layers, two tests were performed with the settings mentioned in the training of the neural network section.

Table 2 shows the effects of training the neural network with and without freezing the layers. The accuracy of a network with frozen layers is 73.0%, and increases to 92.4% when the layers are not frozen. With the layers frozen, the accuracy, precision, recall and f1 score are 73.0%, 68.5%, 59.2% and 56.7%, respectively. With full learning, the accuracy improves to 92.4%, precision to 88.6%, recall to 88.6% and f1 score to 88.7%.

### 3.3. Synthetic Noise

Attempts at synthetic to real transfer [36,44] use Gaussian noise as a data augmentation on their training dataset. However, they do not explicitly test the effectiveness of adding the noise. Furthermore, Ref. [37] found the effect of noise to be negligible. We have found that adding Gaussian noise to the training dataset improves the accuracy of the neural network model when testing on the real-life dataset.

Table 3 shows the effects of adding Gaussian noise as an augmentation to the training dataset. With no noise added, the accuracy, precision, recall and f1 score are 79.4%, 76.0%, 69.0% and 67.5%, respectively. With Gaussian noise added as an augmentation to the training dataset, the accuracy improves to 92.4%, precision to 88.6%, recall to 88.6% and f1 score to 88.7%. This is in line with the findings of [36], who found that adding noise to the training dataset improved the accuracy of their results.

To explain the effects of adding noise to the dataset, Ref. [45] visualizes the effect of noise on neural networks by using sensitivity maps. This method aims to find pixels that strongly influence the final decision. It shows that adding noise to the training dataset provides a de-noising effect to the sensitivity map. To test if the same effect can be seen on our neural network model, we have used a PyTorch implementation of the SmoothGrad visualization method https://github.com/pikahhh/pytorch-smoothgrad (accessed 27 March 2022).

**Figure 3:**
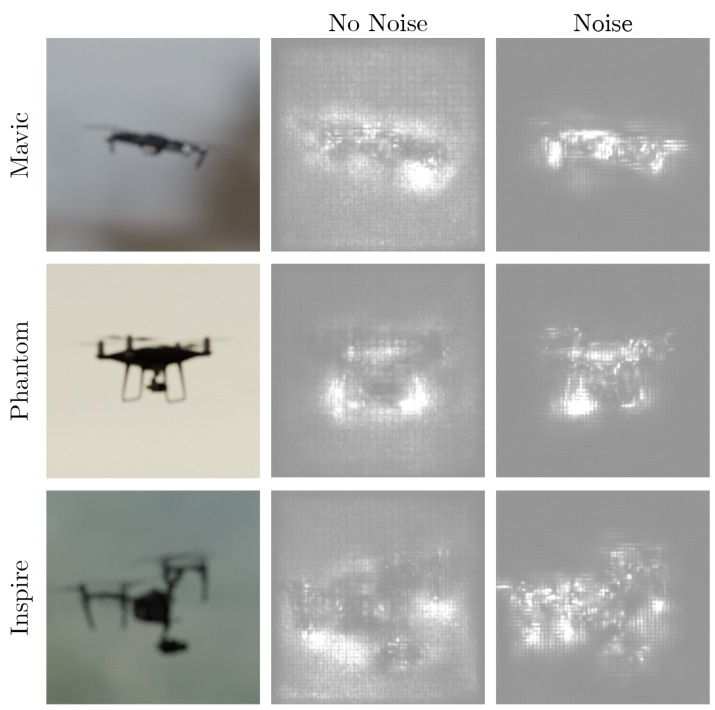
SmoothGrad visualizations of the neural network models trained on a dataset with and without Gaussian noise. The drone image input into the network is shown in the left column. The middle column shows the SmoothGrad visualizations of the CNN trained on a synthetic dataset with no Gaussian noise added. The column on the right shows the SmoothGrad visualizations of the CNN trained on a synthetic dataset with Gaussian noise added. Qualitatively, it appears that, by adding Gaussian noise, more pixels in the area covering the drone are activated. In contrast, when noise is not added, more pixels in the background are activated.

Figure 3 shows the SmoothGrad visualization. The left column shows the input image, the middle column shows the sensitivity map for the neural network trained on a dataset with no noise added as an augmentation and the right column shows the sensitivity map of the neural network trained on a dataset with noise added as an augmentation. The sensitivity maps shown are an average of 50 generated sensitivity maps. The top row shows a visualization of a synthetic DJI Mavic, the middle row of a synthetic DJI Phantom and the bottom row of a synthetic DJI Inspire. The difference between the no noise and noise visualizations is that, in the no noise column, the activated pixels are mostly in the background of the object. In contrast, when noise is added to the training dataset, fewer pixels are visible in the background and more pixels are activated in the area covered by the drone. This is consistent with the findings of the Smoothgrad studies. Although this is purely a qualitative analysis, it helps to explain the reason for the quantitative improvement in accuracy shown in Table 3.

### 3.4. Data Augmentations

The images are resized to 256 × 256 pixels and then cropped to 224 × 224 pixels. A random horizontal flip is applied. The image is then transformed into a tensor and normalized. Lastly, the Gaussian noise of mean 0.75 and standard deviation 0.75 is applied to 75% of the images in the dataset. During validation and testing, the Gaussian noise is not added.

### 3.5. Benchmarking Neural Architectures

To find the best performing architecture for this problem, a benchmark is performed by using open-source implementations in PyTorch [39]. The settings described at the beginning of Section 3 are used to train each of the models. As described in Section 3.2 and Section 3.3, the layers are not frozen and Gaussian noise is added as an augmentation. Because the models are sensitive to the learning rate used, each architecture is trained three times using the learning rates of: 0.01, 0.001, and 0.0001. The architectures are described as follows:

ResNets [46] use residual blocks and skip connections to learn the residual mapping. They have been shown to perform better than plain neural networks, especially for deeper networks. The architecture was the winner of the ILSVRC 2015 for image classification, detection and localization. We test four variations: ResNet18, Resnet50, ResNet50-wide and ResNet101. The number refers to the number of layers in the network. ResNet50-wide is a variation of the ResNet architecture [47], which decreases the depth and increases the width.

DenseNets [48] connect all of the layers together. This architecture was able to beat the state-of-the-art on the benchmark datasets at the time of publication. We test DenseNet121, DenseNet 161, DenseNet 169 and Densenet201. The number refers to the number of layers in the network.

**Figure 4:**
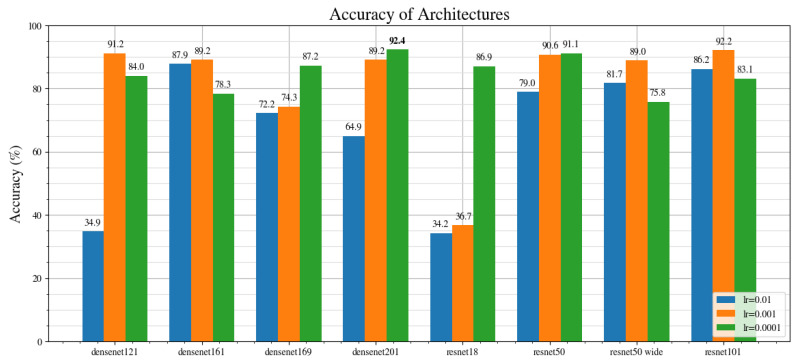
Accuracy of different CNN architectures at different learning rates: 0.01 (blue), 0.001 (orange) and 0.0001 (green).

Figure 4 shows the results of the benchmark. There appears to be no clear trend amongst the architectures, and at an optimized learning rate, they are all able to achieve above 85% accuracy. Densenet201 at learning rate of 0.0001, ResNet101 at learning rate of 0.001 and DenseNet121 at a learning rate of 0.001 are the top 3 performing architectures based on their accuracy score. To further investigate their performance, we can also compare their recall, precision and f1 score metrics.

**Figure 5:**
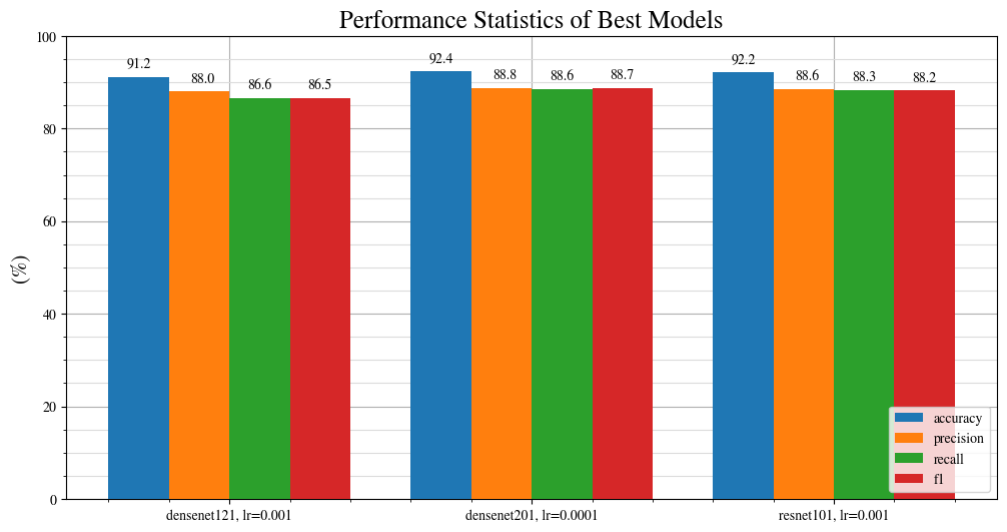
Accuracy, precision, recall and f1 score metrics for the top three performing architectures.

Figure 5 shows the accuracy, precision, recall and f1 metrics for the top three performing architectures in terms of the accuracy metric. Overall, DenseNet201 at a learning rate of 0.0001 shows the best performance across every metric, with an accuracy of 92.4%, precision of 88.8%, recall of 88.6% and an f1 score of 88.7%. It should be noted that, because the DenseNet201 contains more layers, it takes a longer time to train compared with DenseNet121. Thus, although the DenseNet201 performs better overall, the trade-off is that it takes longer to train.

**Figure 6:**
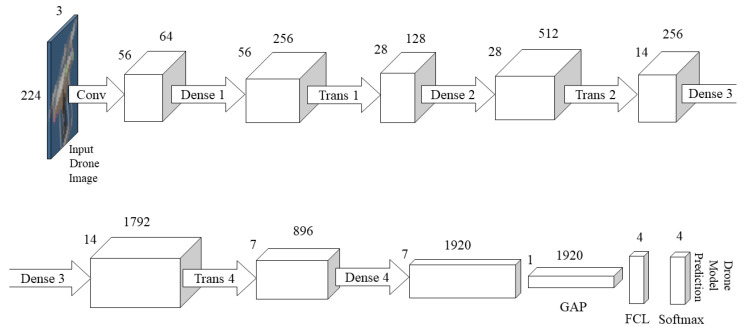
DenseNet201 architecture [48] with the final layer adapted to classify the four classes of DJI Phantom, DJI Mavic, DJI Inspire or no drone. In the graphic, ‘Conv’ refers to a convolutional layer, ‘Dense’ refers to a Dense Block, ‘Trans’ refers to a Transition Layer, ‘GAP’ is the global average pool and ‘FCL’ is the fully connected layer. The input is a 244 × 244 RGB image of a drone, and the output is a prediction of a drone model.

Figure 6 shows the DenseNet201 architecture [48]. The architecture used is the same as in the original paper, with the modification in the final layer to output four classes of drone models: DJI Phantom, DJI Mavic, DJI Inspire and no drone. The input to the CNN is a 224 × 224 RGB image of a drone. The DenseNet201 contains 201 layers. DenseNet architectures connect every layer together. Hence, it has L(L + 1)/2 connections, compared to L connections for standard neural networks. The open-source implementation of the DenseNet201 architecture is available on the GitHub page of PyTorch https://github.com/pytorch/vision/blob/main/torchvision/models/densenet.py (accessed on 27 March 2022).

### 3.6. Ablation Study

To compare which domain randomization parameters have the most influence on the accuracy of the classification, an ablation study is performed. The ablation study is performed by turning off one of the parameters at a time and generating a dataset of 1000 images for training, and 1000 for validation. A neural network model is then trained on this dataset and tested on the real-world Anti-UAV dataset.

**Figure 7:**
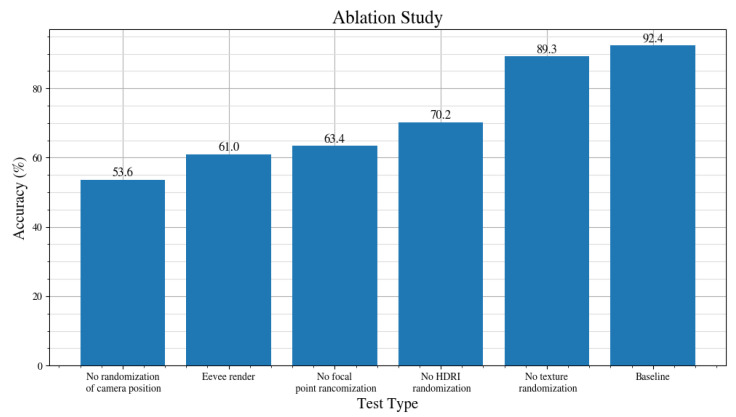
Impact on the accuracy of the neural network by omitting individual randomized components from the data generation procedure described in Section 2.2.

The results of the study are shown in Figure 7. The domain randomization parameters that are compared are:

- **No randomization of camera position** shows a drop in accuracy from 92.4% for the baseline, to 53.6%. In this dataset, the camera is fixed in a single position, as opposed to the camera position being randomized during every frame in the baseline dataset. Because the drone is animated to take off and fly around, the drone still moves. However, this creates a much smaller variation in the orientation of the generated images. This shows that randomizing the position of the camera is the most important domain randomization parameter that is compared in this study.
- **Eevee render** engine shows a drop in accuracy from 92.4% for the baseline, which uses the Cycles render engine, to 61.0%. The difference between Eevee and Cycles render engines is that Eevee is a real-time engine and works similarly to game engines. Cycles is a ray-tracing engine that simulates the physical behaviour of light. It creates more realistic renders but is slower to generate them. The result of the Cycles engine performing better than Eevee is not surprising—the datasets are of the same size. However, the trade-off of cycles is that it is slower to generate renders.
- **No focal point randomization** shows a drop in accuracy from 92.4% for the baseline, to 63.4%. The images generated using this method produce images perfectly focused on the object, with sharp features of the drone. With focal point randomization enabled, the focus point of the camera is not always at the same distance as the drone, which produces blurry images of the drone. The reason for enabling this randomization is that the images of drones filmed in real-life are seldom focused on the drone. This is because it is hard to focus on a small drone that may be hundreds of metres away from the camera. Hence, datasets of real-life images of drones, such as the Anti-UAV dataset [20], tend to be blurry.
- **No HDRI randomization** shows a drop in accuracy from 92.4% for the baseline, to 70.2%. In this scenario, all 1000 images in the dataset use the same HDRI. In contrast, with HDRI randomization enabled, the HDRI is changed every 200 frames, meaning five different HDRI scenes are used.
- **No texture randomization** shows a relatively small drop in accuracy from 92.4% from the baseline, to 89.3%. This suggests that randomizing the texture is not as important as randomizing the other parameters. With this parameter enabled, the texture of the drone is randomized every 200 frames to an unrealistic texture. With it enabled, the default colour of the drone is used.

### 3.7. Dataset Size Study

The advantage of the synthetic dataset is that it allows for the generation of a large number of images quickly. To test if increasing the dataset increases the performance of the model on the real-life test dataset, a dataset size study is performed. The DenseNet201 architecture with a learning rate of 0.0001 is used. Noise is added to the training dataset and the layers are frozen.

**Figure 8:**
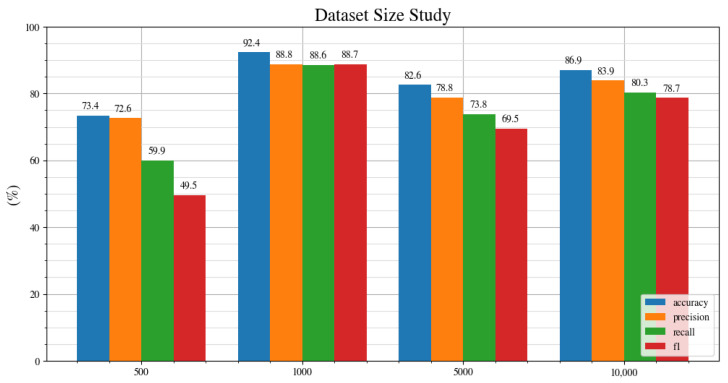
Dataset size study for datasets of 500, 1000, 5000, and 10,000 images.

Figure 8 shows the accuracy, precision, recall and f1 score for the neural network trained on different dataset sizes. The performance is the worst for the smallest, 500 image dataset. It then improves and performs best for the 1000 image dataset. For the larger sized datasets of 5000 and 10,000, the performance drops. This is an interesting result because it is expected that a larger dataset will produce better performance. This leads to the question of: why does the model perform worse when trained on a larger dataset? Ref. [36] found that, after using a dataset size of 10,000 images, the average precision saturates around 79 for the pre-trained network. However, in the dataset study presented in Figure 8, the drop in performance is significant between the 1000 image dataset and the 5000 image dataset. The accuracy drops from 92.4%, and the f1 score drops from 88.7% to 69.5%. This suggests that, unlike in [36], where the performance saturates, the performance drops off.

**Figure 9:**
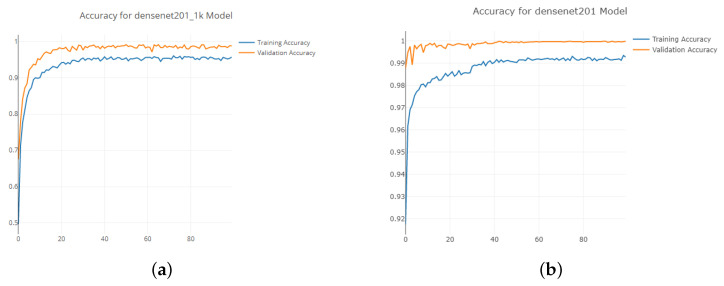
Training accuracy plots across 100 epochs. (**a**) 1000 image dataset, (**b**) 5000 image dataset.

The plots in Figure 9 show the accuracy during the training across the 100 epochs. Both of the plots show the training converging to a very high accuracy. The 5000 image dataset in Figure 9b shows a convergence to near 100% accuracy on the validation dataset. This is a sign of overfitting, suggesting that the network has optimized for the validation dataset too well. In turn, it performs worse on the real-world dataset. This could be potentially solved by adding more variability to the validation dataset.

## 4. Discussion

The best performing model in Section 3 is the DenseNet201 model, pre-trained on the ImageNet dataset, with layers not frozen, with noise added to the synthetic training images, trained on a dataset of 1000 images, using a learning rate of 0.0001, and a batch size of 32.

**Figure 10:**
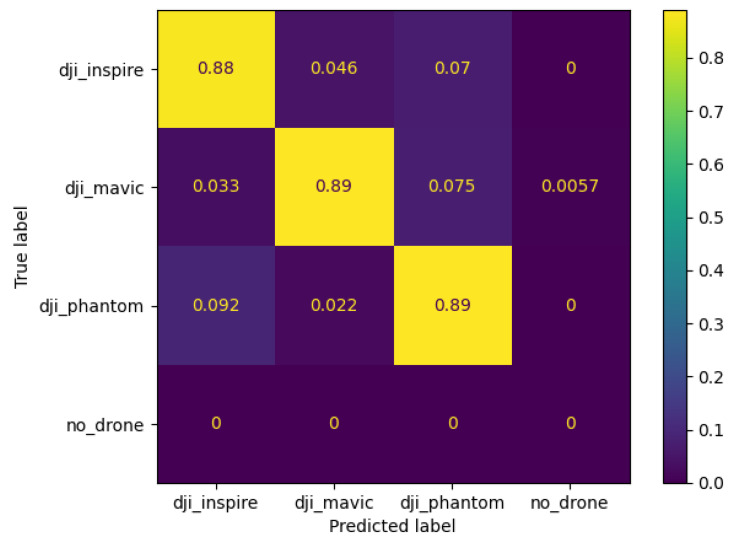
Confusion Matrix of the best performing model.

Figure 10 shows the confusion matrix of the best performing model when tested on the real-life Anti-UAV dataset. The predicted values are shown on the *x*-axis, and the ground truth is shown on the *y*-axis. A true positive is indicated when the predicted value matches the ground truth. The confusion matrix shows that the neural network model correctly identifies the DJI Inspire in 88%, the DJI Mavic in 89%, and the DJI Phantom in 89% of the test images. This shows that the model is not biased towards detecting a single drone type. The confusion matrix presents an overall accuracy of 92.4%, a precision of 88.8%, a recall of 88.6%, and an f1 score of 88.7%. Note that there are no true occurrences of the no drone class in the confusion matrix. This is because there are no no-drone occurrences in the Anti-UAV dataset. Although it is possible to add other occurrences of no-drone images, this is not the aim of this study.

Classification of drones poses a problem because there is not a direct state-of-the-art benchmark to compare this against. Most of the other publications focus on the detection aspect and use region proposal algorithms. In turn, their performance metric, mean average precision, is based on the overlap of the prediction and ground truth. Although this should not be compared directly to the results presented here, we will mention notable results in this category.

Table 4 shows the results of this paper, compared with other literature. Scholes et al. [34] identify the DJI Mavic and DJI Inspire drones. They report an accuracy of 100% on their synthetic dataset, and an accuracy of 100% when they tested on a real-life video of the DJI Mavic taken by a Quantic4x4 camera. For the classification and orientation detection, they use a decision tree coupled with an ensemble network. They then use a U-net type architecture for the segmentation. The difference between theirs and our results is that their focus is on the segmentation of the parts of the drone and on finding the orientation. The results presented here focus purely on the identification of the drone model and bridging the simulation to the real-world gap. Because the results are not tested on the same dataset, it is not possible to make the direct comparison in terms of the classification results between the two methods.

Samadzadegan et al. [28] classify the detected objects into three categories: bird, helicopter and multi-rotor. They report an accuracy of 83%. Their reported precision, recall and f1 can be averaged across the three classes giving average precision of 84%, average recall of 83% and average f1 of 83%. However, their test dataset is different, and they classify different objects, hence a direct comparison should not be made to the results presented here.

Lee et al. [29] train a CNN by gathering a dataset of drone images from the internet. They report an accuracy of 91.6% during training. However, they do not report using a validation dataset. When training neural networks, it is recommended to use separate validation and testing datasets to prevent overfitting [49]. If a CNN model is overfitted to the training dataset, it is likely to perform poorly on test datasets. Hence, it is important to use a validation dataset during training, and a separate test dataset for reporting the final results.

Seidaliyeva et al. [31] classifies loaded and unloaded drones and reports a mean average precision of 75.0% using the YOLOv2 object detection model. The authors were unable to find a public dataset of loaded and unloaded drones, so they created their own. The mean average precision for detection methods is calculated differently to the average precision presented in our paper because it relies on the overlap with the ground truth bounding box, so a direct comparison should not be made to our results.

Ward et al. [38] present a Resnet34 CNN used to classify ships. It is trained using both real and synthetic images to predict the class of a ship (barge, cargo, container, or tanker) from overhead satellite images. This method is similar to the method presented in our approach but for a different domain of ship identification. An accuracy of 96.9% is reported when training the CNN on a mix of real and synthetic data. An accuracy of 59.2% is reported when training on purely synthetic data. Comparing this to our results, we presented an accuracy of 92.4% using DenseNet201 and a purely synthetic dataset. These results suggest that our accuracy could potentially be improved by mixing real-world data into the training dataset.

Table 4 does not contain results from non-visual methods. For comparison, the accuracy of RF detection of drones presented in [9] of 99.2% is higher compared with the visual detection approach presented in this paper. However, there are situations where RF sensors are unsuitable for the detection of drones and optical sensors are more appropriate. It is possible to fuse the data from both of the sensors together to produce an even more reliable system. Hence, even though the RF detection works better, the two detection methods should be developed alongside.

## 5. Conclusions and Further Work

We present a CNN trained on a purely synthetic dataset that correctly classifies drone models in real-life video feeds of the Anti-UAV dataset. To the best of the author’s knowledge, it is the first attempt to classify the drone models on the Anti-UAV dataset. It is also a successful attempt at bridging the synthetic to real-world gap in drone imaging. To achieve this, a synthetic dataset is created by applying domain randomization (random positions, orientations, lighting conditions and textures) to the 3D models of the drones. A benchmark identified that the DenseNet201 architecture showed the highest accuracy. We found that adding Gaussian noise to the training dataset increases the performance of the classifier. We believe that this is an initial step in accurately identifying threats posed by different kinds of drones. We have shown that the method of using 3D models to generate synthetic images to train a neural network can be used for drone model detection in real life videos. The use of this method greatly reduces the time taken to generate a dataset for a new drone model. Given different 3D drones, this method could be used to detect different types of drones than the ones already mentioned in this paper.

There are some shortcomings to this method. A computer with a powerful graphics card is required for the generation of the synthetic images. The CNN was tested on only one real life dataset. The CNN should be tested on other datasets to verify how well it transfers to real life drones in different conditions. The challenge here is the comparison between existing publications because of the difference in the objects being classified and the differences in test datasets. However, we have taken many steps to make sure our method of identifying drones is reproducible on real-life datasets. The drone detection field should aim to unify benchmarks to make direct comparisons easier to produce, similarly to the attempts of the UAV detection and tracking benchmark [22].

The work presented here is an initial step in correctly identifying the threat level posed by the drone. As airports face a growing risk of drone intrusions, it is important for airport security teams to correctly identify the level of risk associated with an intruding drone. This method of training CNNs on synthetic images can be expanded to include other drone models, birds and drones with payloads attached. This would greatly reduce the cost associated with including new threats to airports, compared with traditional methods. With further development, it is possible for the CNN presented here to be used as a part of a larger system which detects drones at airports.

As we have shown that synthetic drone images can be used to train neural networks, this method could be expanded to object detectors such as Faster-RCNN. Thus far, we have classified the drone type. However, with the use of object detectors, we would be able to predict the position of the drone in images. The use of synthetic images is particularly interesting because the simulation is able to output a pixel-level mask of the drone’s position.

This method could be expanded to find the orientation and segment individual parts of the drone, similarly to [34]. Other drone models could also be added to the dataset. Different flying objects, such as planes, helicopters and birds, could also be generated using synthetic images. The CNN model presented in this paper could be improved by further optimizing hyperparameters such as learning rate and batch size. It should be tested on other datasets such as the Drone vs. Bird Challenge dataset. It was found that increasing the size of the dataset saturated above 1000 images. It should be understood why this saturation happens, and if fixed, it would enable the use of larger datasets. Drone area in pixels could be compared with the detection accuracy. This would provide a valuable metric in terms of classification capability.

As the field of drone detection is seeing more high-quality publications, it is important for the field to have a common dataset benchmark. The UAV detection and tracking benchmark [22] try to unify existing datasets in terms of detection accuracy. However, the real-world datasets only contain limited information about the position of the drone in the video frame. Further information, such as orientation, payload, pixel-level masks of the individual drone parts, annotations of birds, multiple drones in one frame and drone model labels, would make the field of drone detection more challenging and would enable researchers to directly compare their results on common real-world datasets.

## Figures and Tables

**Table 1:** Hyperparameters used to train the CNN.

Learning Rate	Momentum	Batch Size	Dropout	Epochs
0.0001	0.9	32	0.25	100

**Table 2:** Effect of freezing layers on the accuracy of the neural network.

Settings	Average Accuracy (%)	Average Precision (%)	Average Recall (%)	Average f1 (%)
Frozen Layers	73.0	68.5	59.2	56.7
Full Learning	92.4	88.8	88.6	88.7

**Table 3:** Effect of adding Gaussian noise on the accuracy of the neural network.

Settings	Average Accuracy (%)	Average Precision (%)	Average Recall (%)	Average f1 (%)
No Noise	79.4	76.0	69.0	67.5
Added Noise	92.4	88.8	88.6	88.7

**Table 4:** Comparison of visual drone detection and classification results with literature. One exception is Ward et al., who classified ships instead of drones, but because they used a synthetic dataset, it makes their results a useful comparison.

Paper	Training Dataset	Classification/ Detection	Method	Test Dataset	Classification Accuracy	Detection mAP
This paper	Synthetic	Classification	DenseNet201	Subset of Anti-UAV	92.4%	n/a
Scholes et al. [34]	Synthetic	Classification	Decision tree coupled with ensemble network + U-Net (segmentation)	Real-life drone video	100%	n/a
Samadzadegan et al. [28]	Real	Both	YOLOv4	Real-life drone video	83%	84%
Lee et al. [29]	Real	Classification	CNN	Internet images	91.6%	n/a
Seidaliyeva et al. [31]	Synthetic	Both	YOLOv2	Video of drones, birds and helicopters	n/a	75.0%
Ward et al. [38]	Mixed synthetic and real	Classification (Ships)	ResNet34	Ship satellite images	96.9%	n/a
Ward et al. [38]	Synthetic	Classification (Ships)	ResNet34	Ship satellite images	59.2%	n/a

## Data Availability

The synthetic training and validation dataset is available to download at https://doi.org/10.17862/cranfield.rd.19423925 (accessed on 27 March 2022).

## Abbreviations

The following abbreviations are used in this manuscript:

CNN	Convolutional Neural Network
HDRI	High Dynamic Range Image
RF	Radio Frequency
